# Interaction between drugs and the gut microbiome

**DOI:** 10.1136/gutjnl-2019-320204

**Published:** 2020-05-14

**Authors:** Rinse K Weersma, Alexandra Zhernakova, Jingyuan Fu

**Affiliations:** 1 Department of Gastroenterology and Hepatology, University of Groningen, University Medical Centre Groningen, Groningen, The Netherlands; 2 Department of Genetics, University of Groningen and University Medical Center Groningen, Groningen, The Netherlands; 3 Department of Pediatrics, University Medical Center Groningen, Groningen, The Netherlands

**Keywords:** proton pump inhibition, immunotherapy, drug metabolism, intestinal microbiology

## Abstract

The human gut microbiome is a complex ecosystem that can mediate the interaction of the human host with their environment. The interaction between gut microbes and commonly used non-antibiotic drugs is complex and bidirectional: gut microbiome composition can be influenced by drugs, but, vice versa, the gut microbiome can also influence an individual’s response to a drug by enzymatically transforming the drug’s structure and altering its bioavailability, bioactivity or toxicity (pharmacomicrobiomics). The gut microbiome can also indirectly impact an individual’s response to immunotherapy in cancer treatment. In this review we discuss the bidirectional interactions between microbes and drugs, describe the changes in gut microbiota induced by commonly used non-antibiotic drugs, and their potential clinical consequences and summarise how the microbiome impacts drug effectiveness and its role in immunotherapy. Understanding how the microbiome metabolises drugs and reduces treatment efficacy will unlock the possibility of modulating the gut microbiome to improve treatment.

**Key messages**

- There is a complex bidirectional interaction between commonly used non-antibiotic drugs and the gut microbiome.
- Commonly used drugs such as proton pump inhibitors, metformin, selective serotonin reuptake inhibitors and laxatives influence gut microbiome composition and function.
- Proton pump inhibitor-induced changes in the gut microbiome can lead to decreased colonisation resistance and the development of enteric infections, including *Clostridium Difficile* infections.
- Gut microbiome composition is associated with antitumour response and the clinical efficacy of treatment with immune checkpoint inhibition.
- Gut microbes can contribute to drug efficacy and safety by enzymatically transforming drug structure and altering drug bioavailability, bioactivity or toxicity.
- Insights into how the gut microbiome interacts with commonly used drugs enable interventions to modulate the gut microbiome and optimise treatment efficacy.

## Introduction

In the past decade we have witnessed exciting discoveries linking the composition and function of the human gut microbiome to numerous common diseases and phenotypes. Association studies have documented changes in the abundance of various gut bacteria in individuals with gastrointestinal phenotypes, including inflammatory bowel disease, irritable bowel syndrome and colorectal cancer, and with diseases of other systems and organs, including cardiovascular and metabolic conditions, autoimmune conditions and psychiatric disorders.^1–9^ In addition to association analyses, intervention studies and animal studies have proven not only the association but also the causality of the gut microbiome in relation to several diseases.^10^ Moreover, the influence of intrinsic and extrinsic factors on gut microbiome composition is increasingly being understood.

**Figure 1:**
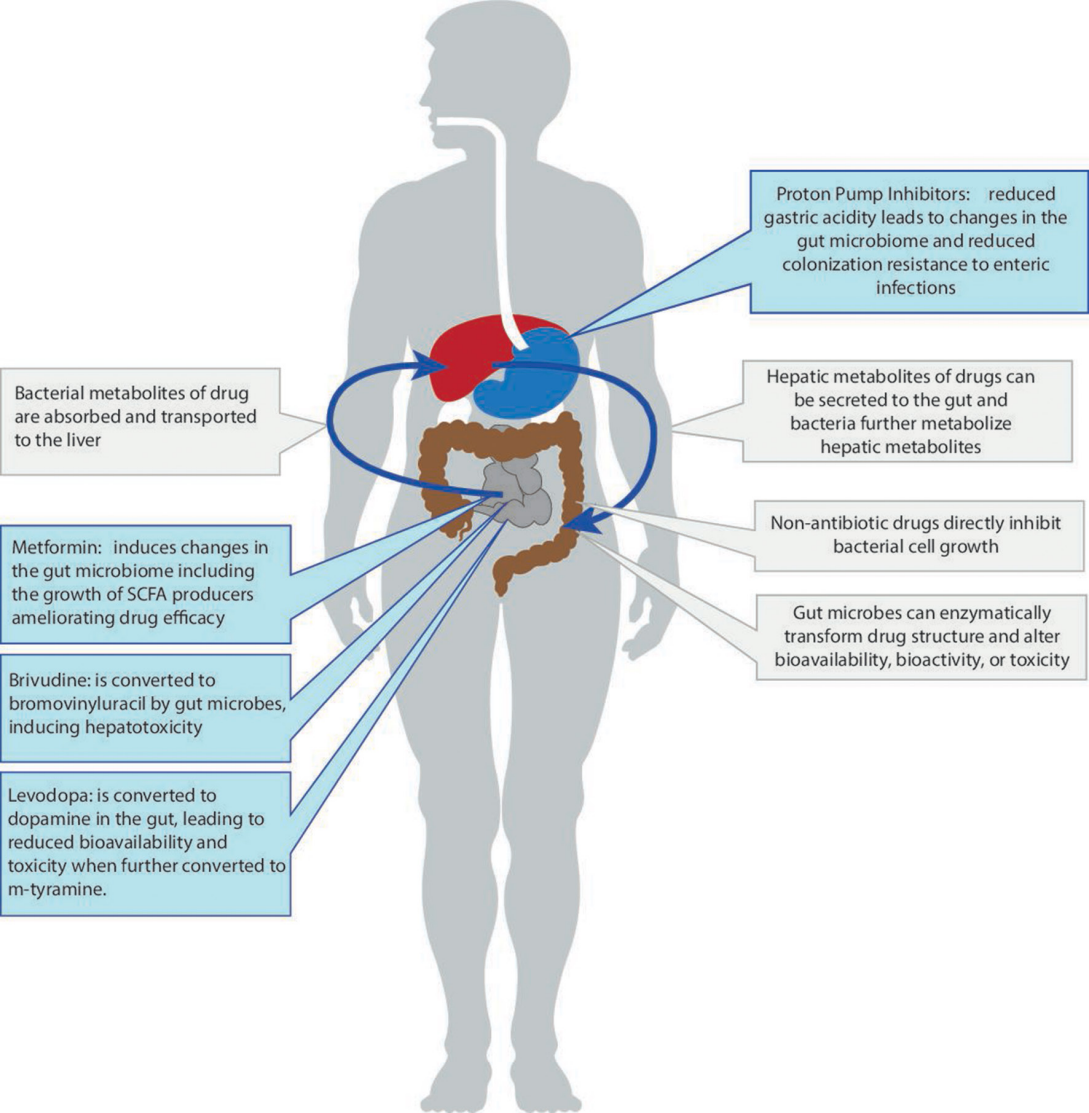
Schematic overview of different interactions between the gut microbiome and commonly used non-antibiotic drugs. SCFA, short-chain fatty acids.

One very important recent finding is that many commonly used non-antibiotic drugs—such as proton pump inhibitors (PPIs) and metformin—change microbiome composition and function.^11 12^ These changes can influence health outcomes (in the case of PPIs) or reduce drug efficacy (in the case of metformin). At the same time, more data has become available showing that the gut microbiome can directly influence an individual’s response to a specific drug by enzymatically transforming the drug’s structure and altering its bioavailability, bioactivity or toxicity—a phenomenon now referred to as pharmacomicrobiomics (figure 1). Finally, the gut microbiome can indirectly impact an individual’s response to immunotherapy in cancer treatment via its influence on the host’s general immune status.^13^ These exciting new insights into the bidirectional interaction between non-antibiotic drugs and the gut microbiome are the focus of the current review.

## Background

### The development of gut microbiome research

Just a few decades ago our ability to analyse the role of the gut microbiome in relation to human health was mainly defined by large technical challenges. Historically, microbiome studies were performed using culturing methods in which one, or a few, bacterial species were isolated and studied in relation to a disease. This research produced numerous important findings, but our ability to analyse other components of the gut ecosystem was limited. The development of the technique to sequence the bacterial 16S ribosomal RNA gene allowed overall taxonomic assessment of the gut microbiome, and this has dramatically increased our knowledge of the broad variations in microbial composition. More recently, whole genome shotgun sequencing, or metagenomic sequencing (MGS), has become a powerful methodology for studying the microbiome. MGS allows identification of not only bacteria, but also viruses, protozoa and fungi, and it enables focussed analysis of bacterial genes and predicted biological pathways. However, as with all sequencing-based methods, MGS results are very dependent on the method used to isolate DNA from stool samples, and this is the major source of the technical variability in the results of microbiome studies.^14^ Other omics approaches, such as metatranscriptomics, metametabolomics and metaproteomics, are also increasingly being used to get a comprehensive picture of the gut ecosystem. Finally, culturomics analysis, which allows deep characterisation of individual associated species and strains, is again becoming an important method to understand the role of specific taxa in relation to diseases.

### Intrinsic and extrinsic factors influencing the gut microbiome

With the aid of next-generating sequencing, gut microbiome analysis has been applied to several human cohorts. One important finding is the large interindividual variability of the gut ecosystem: only a minority of gut microbes are shared across the majority of individuals. For example, in a European data set of 3000 samples, only 17 bacteria were identified as a core microbiome present in >95% of all samples.^15^ The majority of bacteria are rare. Of the 639 species identified in a population study of 1135 Dutch individuals, 469 (73%) were present in fewer than 10 individuals.^16^ This high interindividual variability potentially leads to variations in the metabolic functions carried out by the gut microbiome.

Human cohort-based analysis has further shown that the dynamic nature of the gut ecosystem reflects a complex interaction of the host with lifestyle, dietary, ecological and other factors. Hundreds of intrinsic and environmental factors influence the gut microbiome in healthy individuals, including diet, medication, smoking, lifestyle, host genetics and diseases.^15 17^ Among all environmental factors, commonly used drugs play a particularly important role in the gut ecosystem.

### Association of gut microbiome composition with commonly used drugs in human cohorts

Several human cohort studies have reported associations between use of specific drugs and altered microbial composition and functional profiles (summarised in table 1). One of the first studies to see this was conducted in the Dutch LifeLines-DEEP cohort, and this study reported microbial associations to 19 out of 42 commonly used drugs.^17^ In addition to antibiotics, many human-targeted non-antibiotic drugs were associated with changes in microbial composition. The top microbiome-associated drugs included PPIs, lipid-lowering statins, laxatives, metformin, beta-blockers and ACE inhibitors, and selective serotonin reuptake inhibitor antidepressants, and similar associations were also observed in a Belgium Flemish cohort^15^ and in the TwinsUK cohort^18^ (table 1). It is also worth noting that these drug-microbe associations were mostly assessed for individual drugs. However, we know that patients often take multiple drugs, and this co-medication may be a source of bias when assessing drug-microbe associations. A more recent study further assessed the impact of polypharmacy and comorbidities on the gut microbiome.^19^ This study took a more in-depth look by performing a meta-analysis of the associations between drug use and the gut microbiome in three independent cohorts, including patients with inflammatory bowel disease and irritable bowel syndrome, and found 19 of the 41 medication categories studied to be associated with the gut microbiome. As many of the study participants used multiple drugs, a stepwise approach was used to regress out the effect of polypharmacy. After statistically correcting for polypharmacy, PPIs, metformin, antibiotics and laxatives still showed significant associations with microbial features.^19^

**Table 1:** Effect of common drugs on the microbiome in population studies

Name (analogue UK)	NL% n=1124	UK% n=2737	Effect on alpha div	Effect on beta-div/prop. of core genera	Decreased taxa	Increased taxa
ACE inhibitors	3.91	11.7			s_Dorea_longicatena (1)	g_Rothia (1); g_Blautia (1)
Alpha blockers	0.89	2.73				f_Lactobacillaceae (1); g_Lactobacillus (1); f_Veillonellaceae (1); g_Dialister (1)
Angiotensin-II-receptor antagonists (Sartan)	2.94	6.84		Yes (2)		
Antibiotics (previous month antibiotics)	1.16	6.45	0.45*	Yes (1, 2, 3, 4)	f_Bifidobacteriaceae (1); g_Bifidobacterium (1); s_Bifidobacterium_longum (1); s_Bifidobacterium_adolescentis (1); f_Prevotellaceae (3); f_Peptococcaceae (3); f_Odoribacteraceae (3); f_Clostridiaceae (3); f_Alcaligenaceae (3); f_Anaeroplasmataceae (3); g_unclassified_Lachnospiraceae (4)	f_Enterococcaceae (3); g_Bacteroides (4); g_Oscillibacter (4); g_unclassified_Ruminococcaceae (4)
Antihistamines (H1 inhibitor)	6.14	4.93		Yes (4)	f_Dehalobacteriaceae (3); f_Christensenellaceae (3)	s_Clostridium_bolteae (1)
Beta blockers	5.43	7.42		Yes (1 to 2)	0	f_Streptococcaceae (1); g_Streptococcus (1); s_Streptococcus_mutans (1); g_Rothia (1)
Calcium	1.25	15.7		Yes (1,2)		f_Gemellaceae (3)
Laxatives	1.87	3.19		Yes (1, 2, 4)	g_Collinsella (1); s_Collinsella_aerofaciens (1); f_Lachnospiraceae (1); s_Ruminococcus_obeum (1); g_Coprococcus (1); s_Coprococcus_catus (1); s_Coprococcus_comes (1); g_Dorea (1); g_Faecalibacterium (4)	s_Bifidobacterium_pseudocatenulatum (1); g_Bacteroides (1); s_Bacteroides_stercoris (1); s_Bacteroidales_bacterium_ph8 (1); f_Enterobacteriaceae (1); g_Escherichia (1); g_unclassified_Rhodospirillaceae (4); g_Bacteroides (4); g_Oscillibacter (4); g_Barnesiella (4)
Metformin	1.33	2.9	0.9*	Yes (1, 2, 3)	s_Bacteroides_dorei (1); g_Coprococcus (1); s_Coprococcus_comes (1); g_Dorea (1); s_Dorea_longicatena (1); f_Clostridiaceae (3); f_Ruminococcaceae (3); f_Barnesiellaceae (3); f_Christensenellaceae (3)	f_Streptococcaceae (1); g_Streptococcus (1); f_Enterobacteriaceae (1,3); g_Escherichia (1); s_Escherichia_coli (1)
Opiates (opioid)	1.16	8.58		Yes (3)	f_Dehalobacteriaceae (3);	f_Streptococcaceae (3); f_Micrococcaceae (3); f_Lactobacillaceae (3); f_Eubacteriaceae (3)
Oral contraceptives	10.1	2.61		Yes (2 to 4)		g_Rothia (1)
Paracetamol	0.98	10.6	0.6*	Yes (3)	f_Lachnospiraceae (1); g_Dorea (1); f_Christensenellaceae (3); f_Dehalobacteriaceae (3); f_Oxalobacteraceae (3)	s_Bifidobacterium_dentium (1); s_Streptococcus_salivarius (1); f_Streptococcaceae (3); f_Peptostreptococcaceae (3); f_Eubacteriaceae (3); f_Micrococcaceae (3);
Platelet aggregation inhibitors (aspirin)	2.85	7.83		Yes (1 to 2)	f_Bifidobacteriaceae (1); g_Bifidobacterium (1); s_Bifidobacterium_adolescentis (1)	g_Rothia (1); s_Bifidobacterium_dentium (1); s_Bacteroides_ovatus (1); f_Streptococcaceae (1); g_Streptococcus (1); s_Streptococcus_mutans (1); s_Streptococcus_parasanguinis (1); s_Streptococcus_sanguinis (1); s_Clostridium_bolteae (1); g_Blautia (1); s_Lachnospiraceae_bacterium_3_1_57FAA_CT1 (1); s_Lachnospiraceae_bacterium_7_1_58FAA (1); f_Eubacteriaceae (3)
Proton pump inhibitors	8.27	18.7	8.7*	Yes (1, 2, 3, 4)	s_Eubacterium_hallii (1); s_Eubacterium_ventriosum (1); s_Coprococcus_catus (1); g_Dorea (1); s_Dorea_longicatena (1); f_Ruminococcaceae (1, 3); f_Alcaligenaceae (3); f_Peptococcaceae (3); f_Dehalobacteriaceae (3); f_Coriobacteriaceae (3)	f_Actinomycetaceae (1, 3); g_Actinomyces (1); s_Bifidobacterium_dentium (1); f_Lactobacillaceae (1, 3); g_Lactobacillus (1); f_Streptococcaceae (1, 3); g_Streptococcus (1); s_Streptococcus_anginosus (1); s_Streptococcus_mutans (1); s_Streptococcus_parasanguinis (1); s_Streptococcus_sanguinis (1); s_Streptococcus_salivarius (1); s_Clostridium_bolteae (1); g_Erysipelotrichaceae_noname (1); g_Veillonella (1); s_Veillonella_parvula (1); s_Veillonella_unclassified (1); f_Pasteurellaceae (1, 3); g_Haemophilus (1); s_Haemophilus_parainfluenzae (1); f_Micrococcaceae (3); f_Gemellaceae (3); f_Enterococcaceae (3); f_Fusobacteriaceae (3); f_Enterobacteriaceae (3)
SSRI antidepressants	2.49	6.55		Yes (1, 2, 3)	f_Turicibacteraceae (3); f_Clostridiaceae (3); f_Bifidobacteriaceae (3); f_Peptostreptococcaceae (3); f_.Paraprevotellaceae (3); f_Coriobacteriaceae (3)	
Statins	4.89	25.7		Yes (1, 2, 3)	s_Methanobrevibacter_unclassified (1); g_Coprococcus (1); s_Coprococcus_comes (1); g_Dorea (1); s_Dorea_longicatena (1); f_Peptostreptococcaceae (1); g_Peptostreptococcaceae_noname (1); s_Peptostreptococcaceae_noname_unclassified (1); s_Faecalibacterium_prausnitzi (1)	g_Rothia (1); f_Streptococcaceae (1); g_Streptococcus (1); s_Clostridium_bolteae (1); g_Blautia (1); s_Lachnospiraceae_bacterium_2_1_58FAA (1); s_Lachnospiraceae_bacterium_3_1_57FAA_CT1 (1); s_Coprobacillus_unclassified (1)
Tricyclic antidepressants	0.89	3.77		Yes (1 to 2)	f_Bifidobacteriaceae (1); g_Bifidobacterium (1); f_Streptococcaceae (3); f_Enterobacteriaceae (3); f_Lactobacillaceae (3)	
Vitamin D (cholecalciferol)	1.25	16.5		Yes (1 to 2)		s_Streptococcus_salivarius (1)

Data extracted from four population studies in three populations: Dutch: (1) Vich Vila *et al*, Nat.Communications, 2019,^19^ and (2) Zhernakova *et al*, Science, 2016;^16^ UK: (3) Jackson *et al*, Nat. Communications, 2018^18^ and Belgium: (4) Falony *et al*, Science, 2016.^15^ The table includes drugs used by >2.5% of population in either a Dutch (1) or UK (3) study that showed association to the gut microbiome diversity, composition or taxa. As both Dutch studies (1 and 2) have largely overlapping samples, we only present the taxonomic association results from Vich Vila, which were generated using the more recent MetaPhlAn pipeline and included association on all taxonomic levels.

Name (analog UK): Name of the drug in the Dutch study (1). In brackets, the name of the drug in UK study (3) if another group name is used.

%NL and %UK: Proportion of drug users in the corresponding populations.

Effect on alpha div: Evidence that the drug has an effect on alpha diversity of gut microbiome, * decrease.

Effect on beta-div/prop. of core genera: Evidence that the drug has an effect on beta-diversity or the proportion of core genera (proportion of core genera is only addressed in study 4).

Decreased taxa: Bacterial taxa negatively associated with drug use.

Increased taxa: Bacterial taxa positively associated with drug use.

SSRI, selective serotonin reuptake inhibitor.

Despite the high consistency of drug-microbe associations detected in multiple human cohorts, differences in the estimated effect sizes reflect differences in drug usage in different European countries and in different patients. For instance, the observed impact of antibiotic use on microbial composition in the Belgium Flemish cohort was higher than that in the Dutch LifeLines-DEEP cohort, which is line with the fact that the prescription rate of antibiotics is higher in Belgium than in the Netherlands. Age and gender differences between cohorts also have a strong effect on the frequency of drug usage, and therefore on the observed results.

## Commonly used drugs influencing the gut microbiome

### Proton pump inhibitors

PPIs are among the most commonly used drugs worldwide and are used to treat acid-related disorders such as peptic ulcers, gastro-oesophageal reflux and dyspepsia and for prevention of non-steroidalanti-inflammatory drug-induced gastroduodenopathy and bleeding. Since PPIs are very effective and have a very favourable safety profile, their use has increased very rapidly over the past few decades. In the Netherlands, two million individuals (~12% of the population) now use either pantoprazole or omeprazole by prescription, and similar usage percentages have been reported for other countries such as the UK.^20 21^ The total cost of PPIs in the UK is estimated to be more than £100 million per year.^22^ Moreover, as PPIs are available over-the-counter in the Netherlands, and in many other countries, the total number of PPI users will be much higher than the estimate based on prescriptions alone. In recent years, considerable attention has been paid to the safety profile and potential side effects of chronic use of PPIs. Although the relative risk of adverse drug response (ADR) is low, the high worldwide number of PPI users means that absolute numbers of patients with an ADR can still be high. While there are clear evidence-based indications for the use of PPIs, it has been suggested that up to 70% of PPI prescriptions may be unnecessary,^22^ with use of PPIs as prophylaxis for stress ulcers in patients who do not meet evidence-based prescription criteria a major contributor to this. Another important factor here is that once PPIs are started there is little re-assessment of the original indication for which the PPI was prescribed, and subsequent attempts to stop them lead to unnecessary chronic use.^23 24^

The large population-based study from the Netherlands showed that PPIs were the drugs most associated to a decreased diversity and taxonomical changes in the gut microbiome.^17^ Extending this analysis to include 16 s data from a cohort with inflammatory bowel disease and a cohort with irritable bowel syndrome reproduced these changes across all three cohorts and showed that the relative abundance of up to 20% of bacterial taxa were altered (either decreased or increased) in PPI users compared with non-users.^25^ Similar results showing a lower microbial diversity and lower abundance of gut commensals were observed in a study analysing 16 s data from faecal samples from 1827 twins.^26^ In addition, a small cross-over trial in 12 healthy volunteers showed considerable changes in taxonomy after starting PPIs.^11^

Overall, the taxonomic changes in faecal samples of PPI users show a decrease in abundance of commensal bacteria of the intestine and an increase of bacteria from the oral cavity. These changes include an increase in the families Enterobacteriaceae, Enterococcaceae and Lactobacillaceae and a decrease in Ruminococcaceae and Bifidobacteriaceae, while the shift toward typical oral bacteria is reflected by increases in the species *Rothia dentocariosa* and *Rothia mucilaginosa*, the genus *Actinomyces* and the family Micrococcaceae.^25^ Moreover, it appears that the observed changes are a class-effect of PPIs, since omeprazole, esomeprazole and pantoprazole all showed similar changes. A higher dosage also seems to be associated with larger microbial changes.^19^

A recent study that used metagenomic sequence data, allowing for both high resolution taxonomy and predicted pathway analysis, studied the effect of 41 commonly used drugs on the gut microbiome and again observed that PPIs accounted for the largest number of associations.^19^ After correcting for the impact of concomitant use of other drugs, PPIs were significantly associated with 24 taxa and 133 pathways. The predicted functional changes included the increase of fatty acid and lipid biosynthesis, fermentation nicotinamide adenine dinucleotide (NAD) metabolism, biosynthesis of L-arginine and purine deoxyribonucleoside degradation. These changes in pathways could be explained by the observed changes in the abundance of specific taxa. For example, L-arginine biosynthesis was more prevalent in the microbiome of PPI users. While several bacterial taxa, including *Bifidobacterium* and *Ruminococcus* species, are predicted to contribute to these pathways, statistical analyses showed that only the changes in *Streptococcus mutans* contributed to the predicted pathway changes due to PPI use.^19^ The reduction of gastric acidity induced by PPIs is thought to be responsible for the observed microbial changes since it enables oral bacteria to colonise the gut microbiome, leading to changes in taxonomic homoeostasis (figure 1). This is supported by the observation of an ‘oralisation’ of the gut microbiome in PPI users.^25^ However, an in vitro study assessing the direct effects of commonly used drugs, including PPIs, on gut commensals showed marked changes in bacterial growth rates, implying there is also a direct effect that is potentially mediated through binding of PPIs to bacterial H+/K+ATP ases.^27^

It is important to recognise that PPI-induced changes in the microbiome might actually be contributing to clinically important diseases. For example, previous studies defined changes in the gut microbiome that lead to a decreased colonisation-resistance to enteric infections, including *Clostridium difficile*, *Campylobacter* and *Salmonella*, which are similar to the ones now observed in PPI users.^28 29^ In PPI users, the ORs are estimated to be 1.5 to 1.8 for *C. difficile* and 2.0 to 4.0 for the other pathogenic bacteria.^30^ As it is known that *C. difficile* infections develop in the altered gut microbial environment following the administration of antibiotics,^31^ this could potentially also be true in the setting of PPI use. In addition, PPI initiation and withdrawal influences the clinical course in decompensated liver cirrhosis, potentially through changes in the gut microbiota.^32^ Finally, increased use of PPIs in early childhood may induce long-term changes in the developing gut microbiome, which can lead to obesity in later life.^33^

While the efficacy and safety profiles of PPIs are still very favourable when they are prescribed for evidence-based indications, the medical community should start to rethink their widespread and chronic use and their over-the-counter availability. We have come to the point where we need to carefully assess the long-term effects of PPI-induced changes in the microbiome in up to one-fifth of the population in western society, a shift that has taken place in a relative short period of time in human history, and specifically examine the effects of changing the developing gut microbiome in early life and its influence on health and disease in later life.

### Metformin

Metformin is an oral blood glucose-lowering compound used in the treatment of type 2 diabetes (T2D). While its exact working mechanism is complex, and not fully understood, metformin does inhibit liver gluconeogenesis, and studies increasingly suggest that some of its beneficial effects are mediated by the gut microbiota.^34 35^ Compared with PPIs, which are used for a wide number of indications or symptoms, metformin is (almost) exclusively used in the setting of diabetes, making it harder to disentangle the effect of the drug on the microbiome from changes in the gut microbiome that are related to the underlying disease. However, this has been done by a landmark study, which showed that previously observed changes in the gut microbiome thought to be driven by the underlying T2D were actually caused by the use of metformin.^12^

In an additional intervention study in healthy volunteers, use of metformin resulted in a change in >80 species compared with a control group receiving placebo. Notably, metformin treatment significantly increase *Escherichia coli* and lowers *Intestinibacter* abundance, which is in line with findings from cross-sectional cohorts that compared untreated patients to metformin-treated patients with T2D.^12 36^ Subsequently, the authors transplanted faecal samples from metformin-treated or placebo-treated donors into germ-free mice and observed lower blood glucose levels in the mice that received faecal samples from metformin-treated volunteers, implying a direct effect of the gut microbiome on blood glucose levels. This effect is thought to be mediated by metformin’s effect on short-chain fatty acid (butyrate)-producing bacteria and the abundance of *Akkermansia muciniphila*, as well as through common biological pathways and genes encoded in different metformin-affected bacteria, for example, metalloproteins or metal transporters. Moreover, it is clinically well known that up to one-third of patients taking metformin report gastrointestinal side effects like diarrhoea, bloating and nausea, and the identified metformin-induced changes, including the increase of virulence factors and gas metabolism genes (mainly derived from an increase of *E. coli* species), can contribute to these side effects.^12 36^

The intertwined relationship between metformin and the gut microbiome shows how a commonly used drug can change the gut microbiome and explain part of the drug’s therapeutic function, as well as some of its side effects. It also emphasises the need to rigorously control for confounders like drug use (including of metformin, antibiotics, PPIs and other drugs) when performing microbiome studies looking at specific diseases or conditions.

### Other commonly used non-antibiotic drugs

Large population-based microbiome studies using cross-sectional data from cohorts from the UK, the Netherlands and Belgium have assessed hundreds of factors, including drug use. In addition to PPIs and metformin, these studies have shown that other commonly used drugs, including laxatives, statins, antidepressants and opioids, can explain some of the variability in gut microbiome composition.^15 18 37^

When considering microbial changes induced by the use of laxatives, one should take into account the fact that intestinal transit time, stool consistency and bacterial quantities (eg, microbial load per sample) all influence microbiome features.^38 39^ For example, increased abundances of *Bacteroides* species are observed in individuals taking laxatives, but also in low consistency stool samples. However, in an independent study of mice exposed to polyethylene glycol (PEG) showed a similar increase in *Bacteroides*. In this study, the induction of mild osmotic diarrhoea by administration of PEG induced long-term changes in the gut microbiome, transient disruption of the mucus barrier and subsequent innate and adaptive immune responses.^40^ After PEG administration, the S24-7 Family (within the order Bacteroidales) disappeared and was replaced entirely by outgrowth of the family Bacteroidaceae. This effect was permanent unless the S24-7 Family was replaced. Other taxa, like Verrucomicrobia and Gammaproteobacteria, showed transient changes, but eventually returned to their initial levels. Scarce human data in patients taking bowel preparations have also shown short-term transient changes in microbiome diversity metrics, but detailed studies are lacking.^41^ However, given the findings in mice, it is likely that the use of laxatives has both short-term and long-term effects on gut microbiome composition that are independent of stool consistency and bacterial quantities per sample.

## The gut microbiome influences commonly used drugs

### Emergence of pharmacomicrobiomics

Over the past few decades, pharmacogenomics has become a well-established field that studies how human genome variations affect drug disposition and action. With the increasing recognition of the gut microbiome as the second human genome, the concept of pharmacomicrobiomics has been introduced as a natural expansion of pharmacogenomics. While this field is attracting new attention, the idea that the gut microbiome can impact drug efficacy can be dated back to 1937 and the discovery of the impact of gut microbes on the activation of the antibacterial drug prontosil.^42 43^ Orally administered drugs pass through the upper gastrointestinal track and continue into the intestinal tract, where they encounter the thousands of different species residing in our gut (figure 1). The consequent interactions are bidirectional. On the one hand, drugs can change intestinal microenvironments and affect bacterial growth, composition and function, as described above. On the other, the gut microbiome can directly influence an individual’s response to a specific drug by enzymatically transforming drug structure and thereby altering its bioavailability, bioactivity or toxicity.^44^ Unlike human genetics, the gut microbiome is modifiable, making it an attractive therapeutic target to optimise therapy.

### The bidirectional interaction between drugs and microbes in in vitro and animal studies

In parallel with the gut-microbe associations observed in humans, several in vitro and animal studies have revealed the action modes of drug-microbe interactions and the potential consequences for drug efficacy and safety.

#### Suggested mechanisms for drug impact on the gut microbiome

Drug usage may influence gut microbial compositions in different ways, and at least two action modes have been proposed. The first mode is that drugs can result in the translocation of the microbiome from other body sites to the gut. As described above, PPIs can reduce the acidity barrier of the stomach, which allows oral microbes pass through the stomach to the gut, thereby inducing microbial dysbiosis. The second action mode, which might be the dominant one, is that drugs can change intestinal microenvironments and directly affect bacterial growth. For instance, metformin has been found to promote the growth of short-chain fatty acid producers in the gut, with these bacteria ultimately contributing to the therapeutic effect of metformin in improving insulin resistance and glucose homoeostasis. This second action mode can be bidirectional. Instead of promoting growth of certain bacteria, drugs can also inhibit the growth of specific bacteria, for example, showing antimicrobial activities as antibiotics. This was demonstrated by a landmark high-throughput study^27^ in which the authors systematically assessed the antimicrobial effects of over 1000 drugs, including 835 human-targeted drugs that act by targeting human cells. Notably, 24% of these drugs showed antibacterial activity, affecting the growth rate of at least one of the 40 bacterial strains under study. Among them, eight drugs seemed to be toxic to bacteria, affecting the growth rate of at least 50% of the strains. These drugs included four antineoplastic agents (daunorubicin, 5-fluorouracil, streptozotocin and floxuridine), two anti-inflammatory and antirheumatic drugs (auranofin and diacerein), one antigout drug (benzbromarone) and one drug for peptic ulcer disease (oxethazaine). This result strongly highlights the side effects of cancer therapy on the gut microbiome, as these drugs are often cytotoxic.

#### Suggested mechanism of microbial impact on drug efficacy and safety

It has been shown that gut microbes can contribute to drug efficacy and safety by enzymatically transforming drug structure and altering drug bioavailability, bioactivity or toxicity. For instance, the oral antiviral drug brivudine can be metabolised to bromovinyluracil by both the host and the gut microbiota, with the latter exerting hepatic toxicity. By comparing the plasma and liver concentrations of brivudine and bromovinyluracil between conventional and germ-free mice, Zimmermann *et al* determined that 70% of brivudine toxicity is attributable to gut microbes, particularly to *Bacteroides thetaiotaomicron* and *Bacteroides ovatus*.^45^ The same research group further conducted a systematic analysis to test the metabolic capacity of 76 gut microbial strains on 271 orally administered drugs^46^ and found that 176 drugs (66%) were metabolised by a least one kind of bacterial stain. The drugs that were metabolised by most microbes in vitro included PPIs (pantoprazole, omeprazole and tenatoprazole), the chemotherapeutic drug melphalan, the antimalarial artemisinin and the Parkinson’s drug mesylate. These could be metabolised by almost all the bacterial strains under study. The super drug-metabolising strains were *Bacteroides dorei* (strain DSM17855) and *Clostridium* sp, which could metabolise 164 and 154 drugs, respectively.

**Figure 2:**
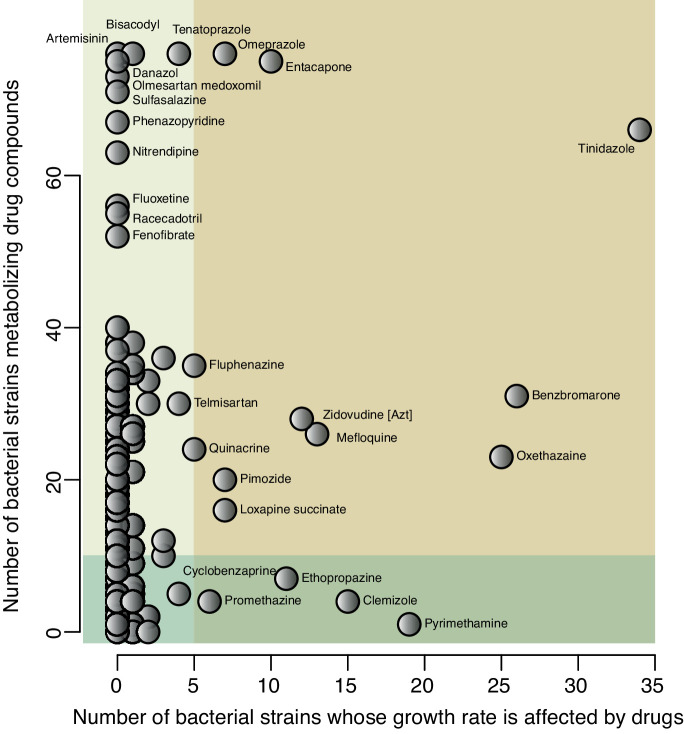
Bidirectional effects of commonly used drugs. X-axis shows the number of bacterial strains (out of 40 strains) whose growth rate has been shown to be affected by a specific drug in vitro. Information extracted from *Maier L, et al Nature 2018;555:623–8* 27. Y-axis shows the number of bacterial strains (out of 76 strains) that can metabolise a specific drug compound in vitro. Information extracted from *Zimmermann et al Nature 2019;570:462–7* 46.

All in all, the results of these two independent studies, one assessing the antimicrobial effect of drugs^27^ and one assessing bacterial ability to metabolising the drugs,^46^ show that sustainable drugs that can function under bacterial metabolism but do not show strong antimicrobial effect (figure 2). However, bidirectional effects are pronounced for several drugs, for example, levodopa.

### The bidirectional effect of the gut microbiome: the example of levodopa

Levodopa, used for the treatment of Parkinson disease, is an intriguing example of microbial impact on drug efficacy. After oral administration, levodopa needs to be absorbed via the small intestine so it can cross the blood-brain barrier and enter the brain, where the human enzyme aromatic amino acid decarboxylase converts levodopa to the therapeutically active dopamine. The bioavailability of levodopa to the brain is a key factor for drug efficacy, and levodopa is often co-administered with catechol metabolism inhibitors, for example, carbidopa and entacapone, to inhibit its off-site metabolism. In recent years, research has shown that the microbial decarboxylases that are part of gut microbial organisms appear to be able to metabolise levodopa. Novel bacterial L-dopa metabolism by tyrosine decarboxylases (tyrDCs) has been identified, dominantly driven by *Enterococcus faecalis*.^47^ Conversely, mutating these tyrDCs in *E. faecalis* can block this bacterial L-dopa-to-dopamine metabolism, thereby improving drug efficacy. In addition to *Enterococcus* species, tyrDCs are also present in *Lactobacillus* species, although *Enterococcus* and *Lactobacillus* species show considerable differences in the efficiency of their L-dopa metabolism.^48^ Moreover, gut bacterial metabolism of L-dopa not only decreases drug availability, it also induces ADRs. *Eggerthella lenta* and 10 other bacterial species were found to contain the dopamine-dehydroxylating enzyme, which can further convert bacterial-derived dopamine to m-tyramine and thereby induce hypertensive crisis.^47^

Interestingly, in vitro, there is also a direct metabolising effect of bacteria on the catechol metabolism inhibitors carbidopa and entacapone that are often co-administered with levodopa. Numerous microbes can metabolise entacapone. *E. faecalis*, for instance, metabolises both levodopa and entacapone at an efficiency of 98.9%.^46^ Conversely, entacapone can also inhibit the growth rate of 10 different species,^27^ including *Ruminococcus torques*, which in turn metabolises entacapone at 84% efficiency.^46^ These results show the complicated bidirectional interaction between drugs and gut microbes.

## The gut microbiome and antitumour response in cancer immunotherapy

**Figure 3:**
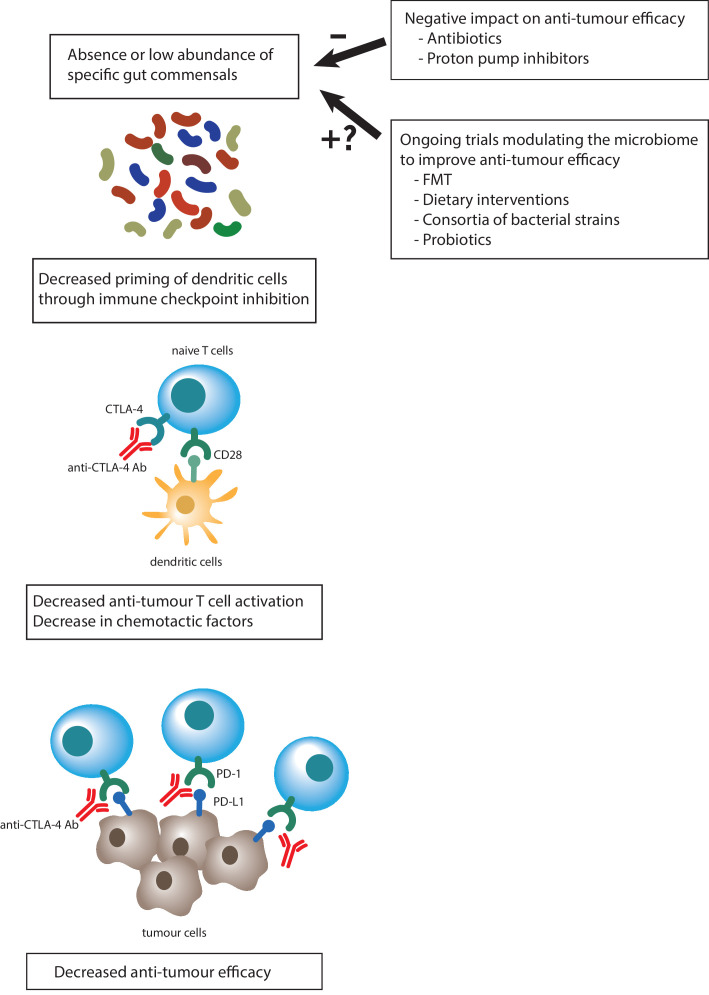
The gut microbiome is involved in modulating the clinical response to cancer immunotherapy. CTLA-4, cytotoxic T lymphocyte antigen 4; FMT, faecal microbiome transplantation; PD-1, programmed cell death protein 1; PD-L1, programmed cell death protein 1 ligand.

We have described how commonly used non-antibiotic drugs can influence the gut microbiome composition and how the gut microbiome composition can influence drug availability and efficacy. In addition, there is also increasing evidence that the gut microbiome is involved in modulating the clinical response to cancer therapy. This occurs specifically in the setting of treatment with immunotherapy using monoclonal antibodies that target programmed cell death protein 1 (PD-1) and its ligand (PD-L1) or the cytotoxic T lymphocyte antigen 4 (CTLA-4).^49 50^ Here the postulated mechanism of action lies in the role of the gut microbiome in fine-tuning the general host immune status and subsequently in antitumour activation of the immune system on checkpoint inhibition (figure 3).

The first breakthrough studies that provided compelling evidence that the gut microbiome influences tumour response were conducted in mice, and these findings were later substantiated in human clinical and microbiome data. It was observed that the efficacy of anti-CTLA-4 therapy was reduced in germ-free mice and in specific pathogen-free mice that were treated upfront with antibiotics.^51^ The mice were then orally fed the species *B. fragilis,* combined with *Burkholderia cepacia* or *B. thetaiotaomicron,* which induced a Th1-mediated immune response and maturation of intratumorous dendritic cells. This, in turn, yielded improved antitumour reactivity to anti-CTLA-4 therapy. In the following phase, the authors transferred faecal material of patients with high levels of *B. fragilis* into mice, and this indeed resulted in improved antitumour response to anti-CTLA-4 therapy.^51^

At the same time, similar results were published in the setting of another anticancer drug that induces PD-L1 blockade. In this case a higher relative abundance of *Bifidobacterium* species in mice resulted in better efficacy of PD-L1 blockade.^52^ This effect could also be induced by faecal microbiome transplantation or cohousing of mice with beneficial species or administration of *Bifidobacterium*-containing probiotics, again through maturation of dendritic cells and increased T-cell reactivity. A more recent study showed similar beneficial results after supplementation with *A. muciniphila*.^53^ In the human setting, the role of the gut microbiome is further substantiated by the observation that patients treated with checkpoint inhibitors who had received treatment with antibiotics prior, during, or after PD-L1 or PD-1 inhibition had lower progression-free survival compared with patients who had not been treated with antibiotics.^53^

A series of subsequent papers have reported the baseline gut microbial composition and specific taxa associated to response or non-response to checkpoint inhibitors. These included, among many others, *Bifidobacterium longum, Enterococcus faecium* and *B. thetaiotaomicron*, which were positively correlated with response to checkpoint inhibitors, and *E. coli*, which was negatively correlated.^54–57^ It has to be noted that at present there is very little overlap in the associated species reported by different studies, and there are several explanations for this. It could be due to the lack of standardised sampling protocols between studies, the lack of standardised statistical correction for confounding factors and/or low statistical power due to relatively small sample sizes. Larger studies aimed at overcoming these issues are currently underway (eg, ClinicalTrials.gov: NCT03643289).

The precise mechanisms by which the gut microbiome influences immunotherapy response still have to be elucidated. It is thought that the microbiome is partially responsible for general peripheral immune homoeostasis and that microbial antigens induce exaggerated T-cell reactivity, which can support tumour-specific responses. In mouse models it has been shown that both innate and adaptive immune cells exposed to specific gut microbes can infiltrate the tumour microenvironment and produce chemotactic factors like CXCL9, CXCR3, CCR9 and CXCL10, which induce trafficking of immune cells to the tumour site.^53 58^ Another hypothetical mechanism is cross-reactivity between microbial and tumour-associated antigens.^59^ Finally, the gut microbiome can produce metabolites, such as short-chain fatty acids, that can have systemic effects on host immunity.^13^

The finding that the gut microbiome influences clinical responses implies that modulating the gut microbiome could potentially improve, or worsen, survival after treatment with checkpoint inhibitors. Intriguingly, both the use of antibiotics and of PPIs has been associated with shorter survival and disease-free survival after treatment with immune checkpoint inhibitors.^60^ On the other hand, the potential to improve survival has resulted in the initiation of multiple ongoing intervention trials. These include studies analysing the efficacy of dietary interventions, the use of specific probiotics and even faecal microbiome transplantation before initiation of checkpoint inhibition for metastatic cancer. A phase 1/2 trial is ongoing with an oral microbial product (VE800) that contains 11 clonal commensal bacterial strains shown to induce CD8 + T cell responses and invigorate the efficacy of checkpoint inhibition. It is worth noting the exceptional speed at which the identification of these 11 strains—published only in 2018—has led to a phase 1/2 intervention trial in the human setting^58^ (ClinicalTrials.gov Identifier: NCT04208958).

Recognition that the microbiome plays a role in antitumour efficacy is a major scientific breakthrough that has changed our thinking on how to predict and improve cancer immunotherapy. Understanding the underlying mechanisms will be crucial, and results of interventional trials will aid in optimising immunotherapy treatment.

## Conclusions

We have described the complex bidirectional interaction between commonly used non-antibiotic drugs and the gut microbiome and described different examples to highlight specific mechanisms. Clinicians need to be aware that it is not only antibiotics that influence the gut microbiome, non-antibiotic drugs can also change the gut microbiome and ultimately lead to impaired health outcomes. At the same time, the pharmacomicrobiomics field is emerging, and a deeper understanding of how the microbiome metabolises drugs or ameliorates the efficacy of, for example, anticancer treatment, will open up the possibility of modulating the gut microbiome to improve treatment efficacy. Clinical trials are already underway, and their results will influence clinical practice in the foreseeable future.
